# Shift work and the risk for metabolic syndrome among healthcare workers: A systematic review and meta‐analysis

**DOI:** 10.1111/obr.13489

**Published:** 2022-06-22

**Authors:** Piumika Sooriyaarachchi, Ranil Jayawardena, Toby Pavey, Neil A. King

**Affiliations:** ^1^ School of Exercise and Nutrition Sciences, Faculty of Health Queensland University of Technology (QUT) Brisbane Queensland Australia; ^2^ Health and Wellness Unit, Faculty of Medicine University of Colombo Colombo Sri Lanka; ^3^ Department of Physiology, Faculty of Medicine University of Colombo Colombo Sri Lanka

**Keywords:** day work, healthcare workers, metabolic syndrome, shift work

## Abstract

Shift work, defined as work occurring outside typical daytime working hours, is associated with an increased risk for metabolic syndrome (MetS) due to several biological and environmental changes. The MetS refers to the clustering of several known cardiovascular risk factors, including insulin resistance, obesity, dyslipidemia, and hypertension. This systematic review aims to evaluate the literature on the association between shift work and the risk of MetS in employees of the health sector. A systematic search was conducted in PubMed, Web of Science, and Scopus databases using appropriate keywords for studies published before September 1, 2021. Eligible studies were those that compared the prevalence of MetS between day and shift healthcare workers; had a cross‐sectional, case–control, or cohort study design; provided sufficient data for calculating odds ratios or relative risks with 95% confidence intervals; and articles in English. The Joanna Briggs Institute prevalence critical appraisal tool was used for quality analysis. Risk for MetS and related measures of effect size were retrieved from studies for meta‐analysis. Twelve studies met the criteria for inclusion in the review and meta‐analysis. Sample sizes ranged from 42 to 738, and the age range of subjects was between 18 and 65 years. Ten studies demonstrated high methodological quality, while two studies were of average quality. Ten out of 12 studies in the review demonstrated a higher risk in shift workers for developing MetS than day workers. The pooled OR of MetS in shift workers based on 12 studies was 2.17 (95% CI = 1.31–3.60, *P* = 0.003; *I* ^2^ = 82%, *P* < 0.001). Shift workers exhibited more than a twofold increase in the chance of developing MetS in comparison with day workers.

## 1 INTRODUCTION

Shift work is referred to as any schedule that is irregular or outside the normal daytime hours (7/8 a.m.–5/6 p.m.). Shift workers account for nearly 20% of the global workforce.^1^ Shift work is considered indispensable in hospitals, industries, and many other essential services to maintain the continuation of services. Although shift work benefits the community, it can be hazardous both for workers and the community, if the workers' alertness and performance are impaired.^2^ Shift work harms the physical, mental, and social well‐being of employees.^3^ Further, it is associated with several chronic diseases, including obesity,^4^ diabetes,^5^ cardiovascular diseases (CVD),^6^ and metabolic syndrome (MetS).^7^

The MetS is a multiplex risk factor that comprises several risks correlates of metabolic origin.^8^ The key components of MetS are dyslipidemia, raised arterial blood pressure, and dysglycemia, with abdominal obesity becoming more prominent as one of the syndrome's key characteristics.^9^ MetS is related to an elevated risk for type 2 diabetes mellitus (T2DM) and CVD.^10^ Moreover, people with MetS have a 46% higher risk of all‐cause mortality, compared with those without.^11^ Several environmental and socio‐economic factors including age, physical inactivity, rapid nutritional changes, lifestyle, and socioeconomic transitions have been recognized as potential risk elements for MetS.^12^, ^13^

Previous research shows that occupational factors such as shift work,^14^, ^15^ excessive sitting‐time, and sedentary occupations significantly increase the risk for MetS.^16^ Several systematic reviews have discovered a positive relationship between shift work and MetS^7^, ^17^ and a positive dose–response relationship with duration of exposure.^18^ However, all these analyses included employees from different industries, and no study has been conducted up to date to investigate the risk among shift workers in the health sector.

Healthcare workers face a variety of occupational hazards at their workplace which increases *the* incidences of *work*‐related disease.^19^ These include physical, chemical, biological, radiation, reproductive health, psychiatric disorders, the effects of shift work, and violence.^20^ Moreover, the shift work disorder was found to be the most frequent work‐related disturbance in these employees working on shift schedules.^21^ It has also been noted that the number of continuous duty hours that health care personnel are permitted to work is much higher than in other professions making them more susceptible to diseases like MetS.^22^

The prevalence of hypertension among hospital staff was found to be 26%, which was higher than the 22% found among other occupational groups.^23^ A study in the United States reported that physicians were 9% more likely to die from cerebrovascular disorders than other occupations.^24^ Another large cohort study in the United States that followed shift working nurses over 22–24 years found that unhealthy lifestyles combined with a 5‐year increment of night shifts predicted a higher risk of diabetes.^25^ Moreover, health care workers seem to be at greater risk of burnout than others due to a range of occupational stresses, such as emotionally difficult patient encounters, exposure to death and dying, time pressure and work overload.^26^

Shift work is considered essential in the health sector to maintain continuity of service, as many patients require constant medical attention and monitoring. Therefore, an increasing number of healthcare employees are being forced to work irregular hours. However, the results of prior studies comparing the risk of MetS among different occupational groups, have produced mixed results.^27^, ^28^ Hence, it is of utmost importance to examine whether shift work and its associated lifestyle could accentuate the disease risk in healthcare workers who is already vulnerable due to the occupational hazards. The current review aims to summarize evidence on the association between the risk of developing MetS and shift work among employees of healthcare services.

## 2 METHODS

The PRISMA (Preferred Reporting Items for Systematic reviews and Meta‐Analyses) statement guidelines^29^ were followed in reporting this systematic review and meta‐analysis.

### 2.1 Literature search strategy

The literature search was carried out in five stages in PubMed® (U.S. National Library of Medicine, USA), Web of Science [v.5.4] (Thomson Reuters, USA), and SciVerse Scopus (Elsevier Properties S. A, USA) databases for articles published before September 1, 2021. We searched databases using Medical Subject Headings (MeSH) terms when possible or keywords when otherwise appropriate and included (“shift work” OR “Shift Work Schedule” OR “Work Schedule Tolerance” OR “Night Shift” OR “night work” OR “irregular working hours” OR “night duty” AND “Metabolic Syndrome” OR “Dysmetabolic Syndrome” OR “Cardiometabolic Syndrome” OR “Metabolic X Syndrome” OR “Syndrome X" OR “deadly quartet” OR “insulin resistance syndrome” OR “Reaven's Syndrome” AND “health personnel” OR “health care worker” OR “health worker” OR “caregiver” OR “physician” OR “medical staff” OR “nurses” OR “hospital employees” OR “hospital staff”). In the PubMed database, an “advanced” search was conducted utilizing the above MeSH terms and keywords in the article title and abstract. The Web of Science® database was searched with the advanced search operator TS (Title, Abstract, Author Keywords, Keywords Plus) for the above search terms in the article topic. The Scopus® database was searched for the aforementioned terms in article title, abstract, or keywords (Data S1).

The total citations gathered from three databases were pooled in the second step and removed the duplicates. The remaining articles were examined to see if they were eligible, by reading article “title,” “abstract,” and “full‐text” in the third, fourth, and fifth stages, respectively, using the inclusion and exclusion criteria mentioned below. A manual search was conducted to obtain additional articles from reference lists of included articles. The literature search was conducted independently by two authors (PS and RJ) separately to identify the studies, and any discrepancies were resolved by discussion.

### 2.2 Inclusion and exclusion criteria

The inclusion criteria of relevant articles were (1) articles published in English; (2) research comparing a shift worker group to a standard day worker group employed in the healthcare sector; (3) studies reporting the prevalence of MetS in both day and shift workgroups (night work or rotating work covering the period between 12:00 a.m.–5:00 a.m. or reporting as shift work); (4) with cross‐sectional, case–control or cohort study designs, and (5) studies that provide adequate information to calculate odds ratios (ORs) or relative risks (RRs) with 95% confidence interval (CI). Furthermore, conference proceedings, commentaries, editorials, and book chapters/book reviews were excluded. Here the healthcare worker was referred to as someone who provides direct or indirect care and services to the sick and injured, whether as doctors or nurses, aides, helpers, laboratory technicians, or even medical waste handlers.^30^

In addition, individual studies were only eligible if the prevalence of MetS was determined according to one of the following acceptable criteria: (a) American Heart Association/National Heart, Lung and Blood Institute (AHA/NHLBI) criteria,^31^ (b) National Cholesterol Education Programme's Adult Treatment Panel III criteria (NCEP/ATP III),^32^ (c) International Diabetes Federation (IDF) criteria,^33^ or (d) modified IDF and modified NCEP/ATP III criteria.^34^ When studies estimated the prevalence of MetS using two different criteria, prevalence values based on the most recent criteria were taken into analysis.

### 2.3 Data extraction

The following variables were extracted and tabulated by an author (PS): first author, publication year, country, study design, gender and age of the study population, sample size, the definition of the night shift, MetS criteria, number of MetS cases, RR or OR values and covariates used in the adjustment. A second author (RJ) double‐checked the accuracy of extracted data, and discrepancies were resolved by discussion. Data not presented in the original manuscript (ORs), where possible were calculated using available data.

### 2.4 Assessment of quality

The study quality assessment was done by two independent investigators using the Joanna Briggs Institute (JBI) prevalence critical appraisal tool.^35^ The JBI quality assessment tool has nine components to assess the overall quality of prevalence studies, including sampling procedure, research subjects, data collection, and classification. The presence of these components can be answered either with a yes, no, unclear, or not applicable. The total number of “yes” responses was counted for each study. A higher number of “yes” responses indicated a lower risk of bias.

### 2.5 Data analysis

The meta‐analysis comprised the studies that matched the eligibility criteria and reported unadjusted/adjusted ORs or RR values with 95% CIs or provided enough data to calculate unadjusted ORs at 95% CIs. The most adjusted model value was used in our meta‐analyses if a publication reported the results of many adjusted ORs or RRs. In addition, we pooled the effect estimates separately, for unadjusted and adjusted ORs. A subgroup analysis was performed according to the occupation.

The *I* ^2^ statistical test was used to examine the heterogeneity among studies. Initially, we performed a fixed‐effects meta‐analysis, but if *I* ^*2*^ was large (>60%), which suggests substantial heterogeneity between studies, a random‐effect model was used.^36^ A *P* value of 0.05 or lower was considered statistically significant. The between‐study variance was represented by Tau.^2^ A funnel plot was constructed to evaluate publication bias by visual assessment. The analysis was performed with Rev Man version 5.3 statistical software package using the generic inverse variance method.

## 3 RESULTS

**FIGURE 1:**
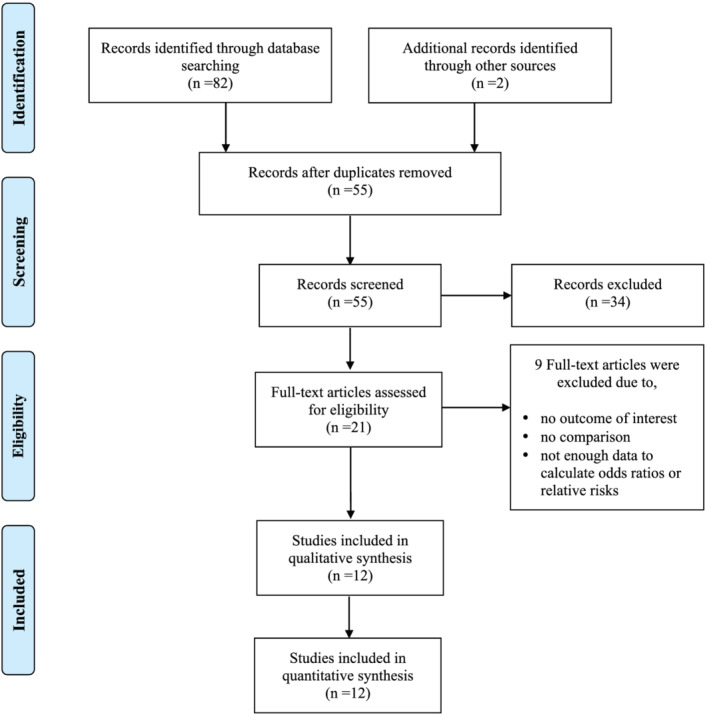
Preferred reporting items for systematic reviews and meta‐analyses flow diagram for study selection

The literature search was performed according to the aforementioned search criteria and the search strategy is summarized in Figure 1. The search resulted in 82 citations (PubMed: 19; Web of Science: 36; SciVerse Scopus: 27), while two additional articles were identified from search of references. The database search resulted in 55 articles after removing duplicates. After initial screening, based on titles and abstracts, 21 articles were selected for further full‐text review. After screening for inclusion and exclusion criteria, 12 studies were included.

A summary of the included articles is presented in Table 1. Out of the 12 eligible articles identified, six studies were conducted in North and South America,^37^, ^40^, ^42^, ^49^, ^50^, ^51^ two in the Middle East,^43^, ^45^ two in Europe,^39^, ^48^ and two in Asian countries.^38^, ^46^ The review included 11 cross‐sectional studies and one cohort study.^39^ The sample sizes ranged between 42 and 738 workers, while the age range of the participants was between 18 and 65 years. Only female workers were involved in five studies,^37^, ^40^, ^46^, ^50^, ^51^ whereas the others included both males and females. Night shift work was defined in five studies in terms of frequency and time range,^39^, ^43^, ^48^, ^49^, ^50^ while one study simply reported only the time range.^46^ Other studies did not give a clear definition. All the night shift definitions covered the time period from 11:00 p.m. to 5:00 am.

**TABLE 1:** Characteristics of the included studies

Author, published year, country	Study design	Gender; sample size; age	Occupation	Sample size		Definition of shift work	MetS criteria	MetS cases (%)		*p* value	OR/RR	Adjusted confounders
				Day	Non‐day			Day	Night		Adjusted/crude	
Arias et al.,^37^ 2021; Ecuador	CS	M & F 300 18–60 years	Volunteers worked in healthcare centers	150 (M:6; F:90)	150 (M:46; F:104)	8 h or more at night, from 7:00 p.m. to 6:00 a.m. and ≥6 month	NCEP‐ATP III	6 (4.0%)	84 (56.0%)	<0.001	22.13 (8.68–66.07)^c^	Age, gender, physical activity and energy consumption.
Copertaro et al.,^38^ 2008; Italy	CS	M & F 147 (M:74, F:73) 35–65 years	hospital staff (mostly nurses)	77 (M:31; F:46)	70 (M:43; F:27)	10:00 p.m.–6:00 a.m. night shift and ≥1.5 nights/month	IDF	16 (20.8%)	26 (37.1%)	<0.05	2.25 (1.08–4.69)^b^	None
Farha and Alefishat,^39^ 2018; Jordan	CS	M & F 140 (M:60; F:80) 20–59 years	Hospital employees	58 (M:13; F:45)	82 (M:47; F:35)	4:00 p.m. to 7:00 a.m. ≥4 night shifts/month for ≥3 years	AHA/NHLBI 2005	6 (10.3%)	13 (15.9%)	0.48	1.63 (0.58–4.58)^b^	None
Holanda et al.,^40^ 2018; Brazil	CS	F 42 26–40 years	Nursing professionals	12	30	≥3 h between 10:00 p.m. to 5:00 a.m. for ≥6 months.	IDF	4 (33.3%)	12 (40.0%)	0.24	1.33 (0.33–5.43)^b^	None
Jung et al.,^41^ 2020; Korea	CS	F 403 20–45 years	Nurses	162	241	11:00 p.m. to 7:00 a.m.	NCEP‐ATP III	40 (24.7%)	47 (19.5.0%)	NR	0.74 (0.46–1.19)^b^	None
Kirk et al.,^42^2015; Canada	CS	F 466 45.8 ± 9.4 years	Hospital employees	300	166	NR	JIS	60 (20.0%)	38 (22.9%)	NR	1.30 (0.78–2.12)^c^	Age, personal, and leisure time physical activity
Korsiak et al.,^43^ 2018; Canada	CS	F 294	Hospital employees. (mostly nurses—68%)	152	142	NR	JIS	18 (11.8%)	29 (20.4%)	0.04	2.72 (1.38–5.36)^c^	Age
Kumar et al.,^44^ 2021; India	CS	M & F 160 (M:80; F:80) 25–50 years	Hospital staff	80 (M:46; F:34)	80 (M:46; F:34)	NR	IDF	33 (41.3%)	20 (25.0%)	0.03	0.55 (0.24–1.29)^c^	Age, gender, diet, physical activity, sleep, stress, alcohol consumption
Lajoie, et al.,^45^ 2015; Canada	CS	F 271	Hospital employees	150	121	NR	JIS	20 (13.3)	27 (22.3)	0.05	2.29 (1.12–4.70)^c^	Age, household income, menopausal status
Niazi et al.,^46^ 2018; Iran	CS	M & F 410 (M:124; F:286) 33.43 ± 8.51 years	Nurse, emergency workers, office workers, and others	56	352	NR	NCEP‐ATP III	NR	NR	NR	3.97 (1.67–9.45)	None
Pietroiusti et al.,^47^ 2010; Italy	Cohort	M & F 738	Nurses	336 (M: 92; F:244)	402 (M:124; F:278)	Between 9:00 p.m. and 7:00 a.m. and 4 nights/month for ≥1 year	Updated NCEP‐ATP III	6 (1.8%)	36 (9.0%)	NR	5.10 (2.15–12.11)^a^ ^,^ ^c^	Age, gender, smoking, alcohol intake, familiar history, physical activity
Ritonja et al.,^48^ 2018; Canada	CS	M & F	Hospital employees	160	166	NR	JIS	19 (11.9%)	35 (21.0%)	<0.01	1.98 (1.08–3.64)^b^	

Abbreviations: AHA/NHLBI, American Heart Association/National Heart, Lung and Blood Institute; CS, cross‐sectional; F, female; h, hours; IDF, International Diabetes Federation; JIS, joint interim statement; M, male; NCEP ATP III, National Cholesterol Education Program, Adult Treatment Panel III; NR, not reported; OR, odd ratio; RR, relative risk.

^a^ Relative risk.

^b^ Calculated odd ratios with MetS cases in day and night shift worker.

^c^ Adjusted odd ratios.

All the studies except the study by Niazi et al.^45^ reported the prevalence of MetS among day and shift employees separately. In these studies, the prevalence or incidence of MetS among day workers ranged from 1.8% to 41.3%, whereas it ranged from 9.0% to 56.0% among shift workers when assessed using the aforementioned criteria. Out of the 12 studies, 10 studies reported a higher prevalence of MetS among shift employees compared with day employees, with the difference being statistically significant in five of them (*P* ≤ 0.05). Only the two studies by Kumar et al.^38^ and Jung et al.^46^ reported contradictory results.

Six studies reported adjusted OR or RR values for the effect of shift work on MetS,^37^, ^38^, ^39^, ^40^, ^49^, ^51^ while one study reported the crude value.^45^ For the remaining five studies, crude OR values were manually calculated.^42^, ^43^, ^46^, ^48^, ^50^ The study by Pietroiusti et al.^39^ showed the highest OR value, while the lowest was reported by Kumar et al.^38^ Age was the most commonly adjusted covariate in all of the aforementioned seven studies where effect size was adjusted for confounders. Three studies demonstrated OR adjustments for all three parameters of age, gender, and physical activity levels.^38^, ^39^, ^49^

Results of the quality assessment process are presented in Table S1. The total number of “yes” responses varied from 6 to 9 between studies. Out of the 12 studies in the review, 10 studies appeared to be of high quality with the total number of “yes” scores varying between 7 and 9. Two studies received scores of 6 and seemed to be of average quality. Across all studies, the average number of “yes” responses was 7.42.

**FIGURE 2:**
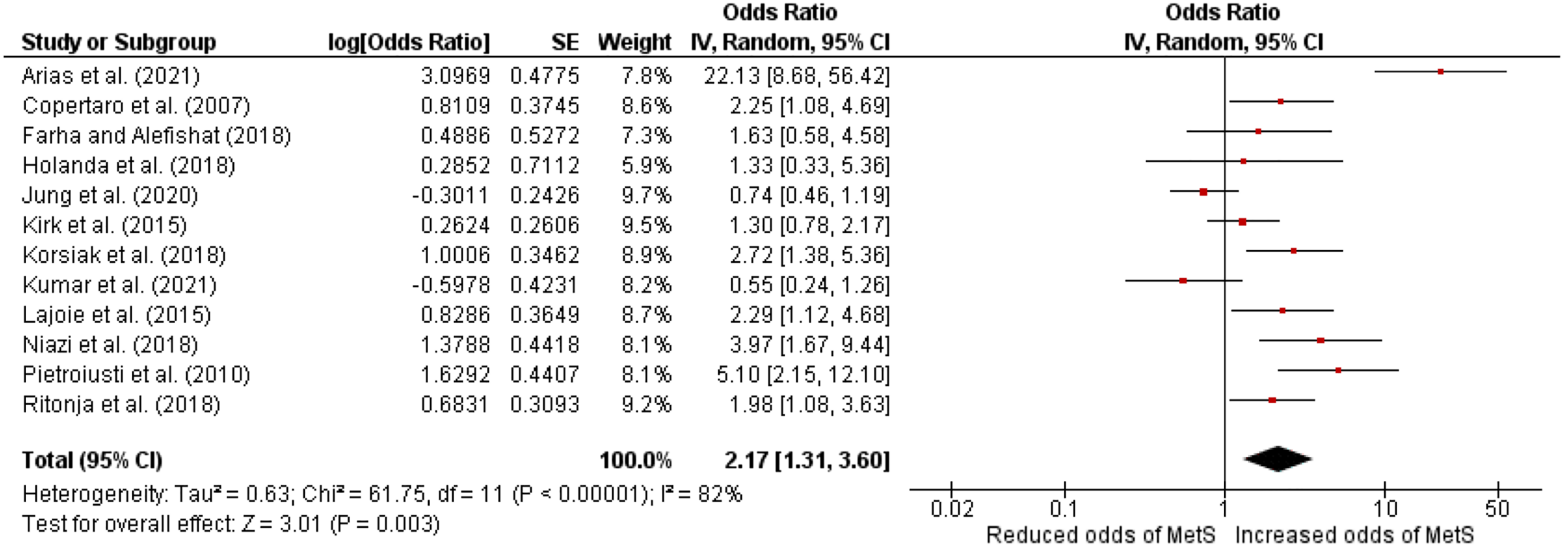
Association between metabolic syndrome and shift work among healthcare workers

All 12 articles were included in the quantitative analysis. A random‐effects analysis was adopted because high *I* ^2^ values from fixed‐effects analysis suggested significant heterogeneity between studies. The meta‐analysis results revealed that the odds of developing MetS were significantly greater in shift employees than in day employees with a pooled OR value of 2.17 (95% CI = 1.31–3.60, *P* = 0.003; *I* ^2^ = 82%, *P* < 0.001) (Figure 2). Since the study by Arias et al. had a large OR and so acted as an outlier, the meta‐analysis was reconducted with the outlier removed, but the pooled effect size remained significant (OR 1.77; 95% CI = 1.19–2.64, *P* = 0.005; *I* ^2^ = 70%, *P* < 0.001) (Figure S1). The pooled estimate of unadjusted ORs based on six studies was 1.71 (95% CI = 0.98–2.97, *P* = 0.06; *I* ^2^ = 67%, *P* < 0.009) (Figure S2). When adjusted ORs were analyzed, the pooled estimate based on six studies was 2.74 (95% CI = 1.15–6.51, *P* = 0.02; *I* ^*2*^ = 88%, *P* < 0.001) (Figure S3). Based on the subgroup analysis for nurses, the pooled adjusted OR (95% CI) was estimated as 2.20 (95% CI = 1.11–4.38, *P* = 0.02; *I* ^2^ = 79%, *P* < 0.001) (Figure S4). The funnel plots revealed that the studies were distributed fairly symmetrically around the aggregate effect size, indicating that there was little publication bias (Figure S5).

## 4 DISCUSSION

Although earlier studies have examined the association between MetS and shift work, this is the first to quantify the risk for shift workers in the health sector. The results of the current study were consistent with the meta‐analyses done by Wang et al. and Yang et al., where both found significant positive associations between night shift work and MetS among workers from different industries. In Wang et al., the pooled RR for the association between night shift work and MetS risk was 1.57 (95% CI = 1.24–1.98),^18^ while Yang et al. reported a RR of 1.30 (95% CI: 1.19–1.41).^47^ A similar study among a group of Iranian drivers revealed that the risk of MetS is higher among shift working drivers (OR 1.495; 95% CI: 1.349–1.657).^41^ Our study discovered a greater occurrence of MetS for employees working in the health care sector (OR 2.17; 95% CI = 1.31–3.60, *P* = 0.003). The pooled OR for the adjusted estimates was even higher when the analysis was adjusted for confounding factors 2.74 (95% CI = 1.15–6.51, *P* = 0.02). Therefore, it is evident that health sector workers have a pronounced risk of MetS.

According to recent research, circadian rhythm disruption (chronodisruption) may lead to manifestations of MetS.^44^ The relationship between the circadian system and different MetS components such as impaired glucose and lipid metabolism,^52^, ^53^ adipose tissue function, and cardiac,^54^ vascular, and hemostatic function^55^ has been studied widely. The prevalence of adiposity and MetS is increased by shift employment, sleep deprivation, and exposure to bright light at night.^56^ In line with these findings, a meta‐analysis has demonstrated a significant increase in the body fat percentage of shift employees when compared to the non‐shift group.^57^ Further, among nurses, a correlation has been detected among the variables of anxiety and stress with MetS.^58^

Health sector employees must work non‐traditional shifts which often result in sleep disruption, reduced sleep duration, or drowsiness due to misalignment of the circadian pacemaker with sleep–wake timing.^1^ The progressive decrease in the amount of time spent sleeping, may disturb synchronization between sleep/activity and alternating periods of feeding/fasting and energy storage/usage.^59^ Sleep deprivation is an individual risk factor for the development of MetS and an increased risk of obesity.^60^ Leptin, which is primarily produced by adipose tissue and is the product of the obesity gene, controls food intake and energy expenditure, fatty acid metabolism of skeletal muscle, and hepatic glucose synthesis.^61^ The structure of leptin is similar to that of pro‐inflammatory cytokines like interleukin (IL)‐6, which inhibits insulin activity. In addition, excessive fatty acid outflow from adipose tissue is a key factor in the onset of MetS.^62^ Because insulin regulates this pathway, insulin resistance causes an increase in the free fatty acid release from adipose tissue and poor removal of triglyceride‐rich lipoproteins.^63^ As a result, lipid accumulation in ectopic regions like the liver, skeletal muscle, and pancreatic islets can lead to the organ's functional impairment.^64^

Out of the 12 studies included in the current review, 10 studies demonstrated a positive relationship between shift work and MetS, while the other two studies by Kumar et al.^38^ and Jung et al.^46^ resulted in contradictory outcomes. However, Kumar et al. reported that the OR for MetS among shift workers was not significant after adjusting for confounding variables including age, physical activity, sleep, and diet.^38^ According to Jung et al., young nurses usually work shifts, and shift work nurses caring for patients will participate in more physical activities than non‐shift work nurses doing administrative tasks.^46^

Several limitations are associated with this study. First, the diagnostic criteria for MetS differed between studies, limiting the study's comparability. Second, the inconsistency in the definition of night shift work. Because the night shift duration and frequency per week differed among studies, it could have led to a dilution of the pooled effect during the meta‐analysis. Third, there was considerable heterogeneity among the selected studies relative to sample sizes, primary objectives, and study designs. Finally, the OR values used for the meta‐analysis included both adjusted and unadjusted values. When studies reported adjusted values, we always used the best‐adjusted value for the highest number of covariates to minimize bias during the meta‐analysis. Furthermore, as to literature, the use of OR to compare effect sizes in meta‐analysis is not recommended as the magnitude of odds ratios is easy to misinterpret.^65^ Also it has been reported that odds ratios cannot be interpreted as absolute effects, nor could be compared across different study samples.^66^ The meta‐analysis indicated high heterogeneity which could be due to a variety of factors, including the differences in shift schedules among the workers. However, we could not conduct a subgroup analysis based on different shift schedules due to the lack of sufficient information. Therefore, to better understand these associations more future research is needed, such as appropriately powered cohort studies or interventional clinical trials. Furthermore, conducting subgroup analysis and adjusting for confounding variables, when possible, could reduce heterogeneity and increase precision in future research.

Our results suggest that shift working employees in the healthcare sector are twice as likely to develop MetS compared with their day working counterparts. Although we could not inspect the relationship between years of shift employment and risk for MetS, prior shift work studies have shown a positive dose–response connection with exposure duration.^27^ Therefore, future research should focus on creating intervention measures to reduce the incidence of MetS among shift workers. Research suggests that MetS is caused due to several underlying risk factors, including environmental, behavioral, genetic,^67^ and hormonal variables.^68^ Therefore, future policies and interventions should be planned to change these aspects (Table S2). As body composition and epigenetics are predictors of MetS,^69^ it is recommended that employees be screened at the time of hiring based on their family disease history and BMI to determine their fit for shift work. Further, the physical environment at work plays a vital role in employees' health.^70^ Especially for shift workers, the workplace meal environment, maybe uncomfortable, and canteens may have a limited supply of nutritious food late at night.^71^ Therefore, the availability and accessibility of healthy foods at workplaces should be ensured. Also, the canteens that adhere to healthy dietary guidelines and promote healthy food habits among staff should be promoted. In addition, workplace health promotion programs could be set up to encourage employees to adopt healthy behavioral changes,^72^ such as reducing obesity and increasing physical activity.^73^ Different dietary intervention strategies such as portion control, meal replacements, and energy/calorie‐restricted diets should be introduced to shift working employees. Ultimately the employers and other policymakers should offer more flexible work schedules for shift working employees to ensure their work/life balance.

## 5 CONCLUSION

This is the first meta‐analysis to explore the association between shift employment and the possibility of developing MetS, particularly among the healthcare sector employees. In 10 of the 12 studies in the review, shift workers were shown to have a high prevalence of MetS in comparison to their non‐shift working counterparts. Outcomes of the meta‐analysis showed a twofold increased risk for the development of MetS in shift workers relative to the day group. Therefore, to safeguard shift workers from MetS, health promotion programs as well as other interventional strategies to adopt healthy environmental and behavioral changes should be introduced. In addition, organizations should streamline the shift work system with well‐designed rotational shift schedules to allow employees to maintain work/life balance.

## CONFLICT OF INTEREST

The authors declare that they have no competing interests.

## AUTHOR CONTRIBUTIONS

PS conceived and designed the study. PS and RJ were involved in data collection. PS analyzed data and drafted the manuscript. RJ, NK, and TP critically revised the manuscript. All authors read and approved the final manuscript.

## Supporting information


**Data S1:** Search strategy

**Figure S1.** The pooled estimate of after removing the study Arias et al
**Figure S2.** The pooled estimate of unadjusted ORs
**Figure S3.** The pooled estimate of adjusted ORs
**Figure S4.** Pooled risk estimates of night shift work and metabolic syndrome for nurses
**Figure S5.** Funnel plots to assess publication bias

**Table S1.** Quality analysis

**Table S2.** Recommended policies and interventions to reduce the incidence of MetS among shift workers.
