# Supplementary material for: Shift work and the risk for metabolic syndrome among healthcare workers: A systematic review and meta‐analysis
Source: Obes Rev. 2022 Jun 22;23(10):e13489. doi: 10.1111/obr.13489 (PMC9539605; doi:10.1111/obr.13489)
Supplement: Supplementary file 1 — Data S1: Search strategy [file OBR-23-e13489-s001.docx]

**Data S1. Search strategy**

Example Database Search Terms: Scopus

## ( TITLE-ABS-KEY ( "shift work"  OR  "Shift Work Schedule"  OR  "Work Schedule Tolerance"  OR  "Night Shift"  OR  "night work"  OR  "irregular working hours"  OR  "night duty" )  AND  TITLE-ABS-KEY ( "Metabolic Syndrome"  OR  "Dysmetabolic Syndrome"  OR  "Cardiometabolic Syndrome"  OR  "Metabolic X Syndrome"  OR  "Syndrome X"  OR  "deadly quartet"  OR  "insulin resistance syndrome"  OR  "Reaven's Syndrome" )  AND  TITLE-ABS-KEY ( "health personnel"  OR  "health care worker"  OR  "health worker"  OR  "care giver"  OR  "physician"  OR  "medical staff"  OR  "nurses"  OR  "hospital employees"  OR  "hospital staff" )  AND  LANGUAGE ( English )  AND  ALL ( "humans" )  AND NOT  ALL ( "animals" )  AND NOT  ALL ( "animals and humans" ) )

Other databases searched: Pubmed, Web of Science
