# Supplementary material for: Shift work and the risk for metabolic syndrome among healthcare workers: A systematic review and meta‐analysis
Source: Obes Rev. 2022 Jun 22;23(10):e13489. doi: 10.1111/obr.13489 (PMC9539605; doi:10.1111/obr.13489)
Supplement: Supplementary file 2 — Figure S1. The pooled estimate of after removing the study Arias et al Figure S2. The pooled estimate of unadjusted ORs Figure S3. The pooled estimate of adjusted ORs Figure S4. Pooled risk estimates of night shift work and metabolic syndrome for nurses Figure S5. Funnel plots to assess publication bias [file OBR-23-e13489-s003.pdf]

# Shift work and the risk for metabolic syndrome among healthcare workers: A systematic review and meta-analysis

Piumika Sooriyaarachchi[1, 2*], Ranil Jayawardena[1, 3], Toby Pavey[1], Neil A. King

[1]Queensland University of Technology (QUT), Faculty of Health, School of Exercise and Nutrition Sciences, Brisbane, Queensland, Australia.

[2]Health and Wellness Unit, Faculty of Medicine, University of Colombo, Colombo, Sri Lanka.

[3]Department of Physiology, Faculty of Medicine, University of Colombo, Colombo, Sri Lanka

**Address of corresponding author and e-mail address:**

Piumika Sooriyaarachchi, Queensland University of Technology (QUT), Faculty of Health, School of Exercise and Nutrition Sciences, Brisbane, Queensland, Australia.

E mail: piumika.sooriyaarachchi@hdr.qut.edu.au / piumika.soo@gmail.com

| Study or Subgroup | log[Odds Ratio] | SE | Weight | Odds Ratio IV, Random, 95% CI |
|---|---|---|---|---|
| Copertaro et al. (2007) | 0.8109 | 0.3745 | 9.4% | 2.25 [1.08, 4.69] |
| Farha and Alefishat (2018) | 0.4886 | 0.5272 | 7.2% | 1.63 [0.58, 4.58] |
| Holanda et al. (2018) | 0.2852 | 0.7112 | 5.2% | 1.33 [0.33, 5.36] |
| Jung et al. (2020) | -0.3011 | 0.2426 | 11.6% | 0.74 [0.46, 1.19] |
| Kirk et al. (2015) | 0.2624 | 0.2606 | 11.3% | 1.30 [0.78, 2.17] |
| Korsiak et al. (2018) | 1.0006 | 0.3462 | 9.9% | 2.72 [1.38, 5.36] |
| Kumar et al. (2021) | -0.5978 | 0.4231 | 8.7% | 0.55 [0.24, 1.26] |
| Lajoie et al. (2015) | 0.8286 | 0.3649 | 9.6% | 2.29 [1.12, 4.68] |
| Niazi et al. (2018) | 1.3788 | 0.4418 | 8.4% | 3.97 [1.67, 9.44] |
| Pietroiusti et al. (2010) | 1.6292 | 0.4407 | 8.4% | 5.10 [2.15, 12.10] |
| Ritonja et al. (2018) | 0.6831 | 0.3093 | 10.5% | 1.98 [1.08, 3.63] |
| **Total (95% CI)** | | | **100.0%** | **1.77 [1.19, 2.64]** |

Heterogeneity: $Tau^2 = 0.30$; $Chi^2 = 32.94$, df = 10 (P = 0.0003); $I^2 = 70\%$
Test for overall effect: Z = 2.80 (P = 0.005)

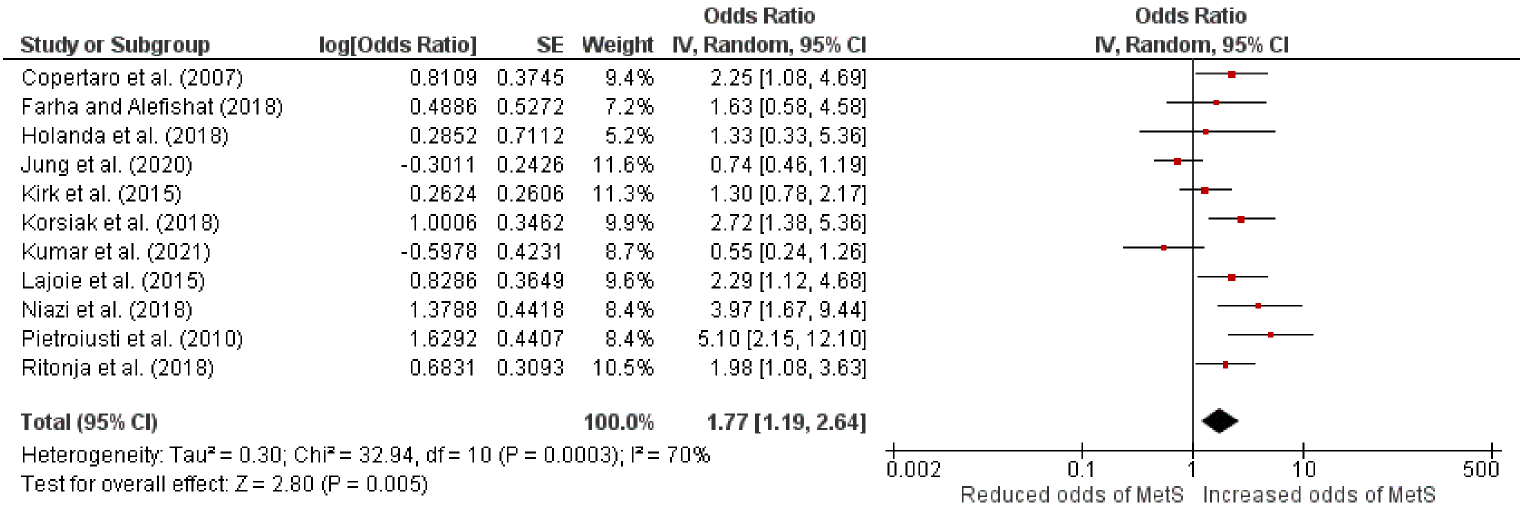


**Figure S1. The pooled estimate of after removing the study Arias et al.**

| Study or Subgroup | log[Odds Ratio] | SE | Weight | Odds Ratio IV, Random, 95% CI |
|---|---|---|---|---|
| Copertaro et al. (2007) | 0.8109 | 0.3745 | 18.1% | 2.25 [1.08, 4.69] |
| Farha and Alefishat (2018) | 0.4886 | 0.5272 | 13.8% | 1.63 [0.58, 4.58] |
| Holanda et al. (2018) | 0.2852 | 0.7112 | 9.9% | 1.33 [0.33, 5.36] |
| Jung et al. (2020) | -0.3011 | 0.2426 | 22.2% | 0.74 [0.46, 1.19] |
| Niazi et al. (2018) | 1.3788 | 0.4418 | 16.1% | 3.97 [1.67, 9.44] |
| Ritonja et al. (2018) | 0.6845 | 0.3097 | 20.1% | 1.98 [1.08, 3.64] |
| **Total (95% CI)** | | | **100.0%** | **1.71 [0.98, 2.97]** |

Heterogeneity: $Tau^2 = 0.30$; $Chi^2 = 15.34$, df = 5 (P = 0.009); $I^2 = 67\%$
Test for overall effect: Z = 1.89 (P = 0.06)

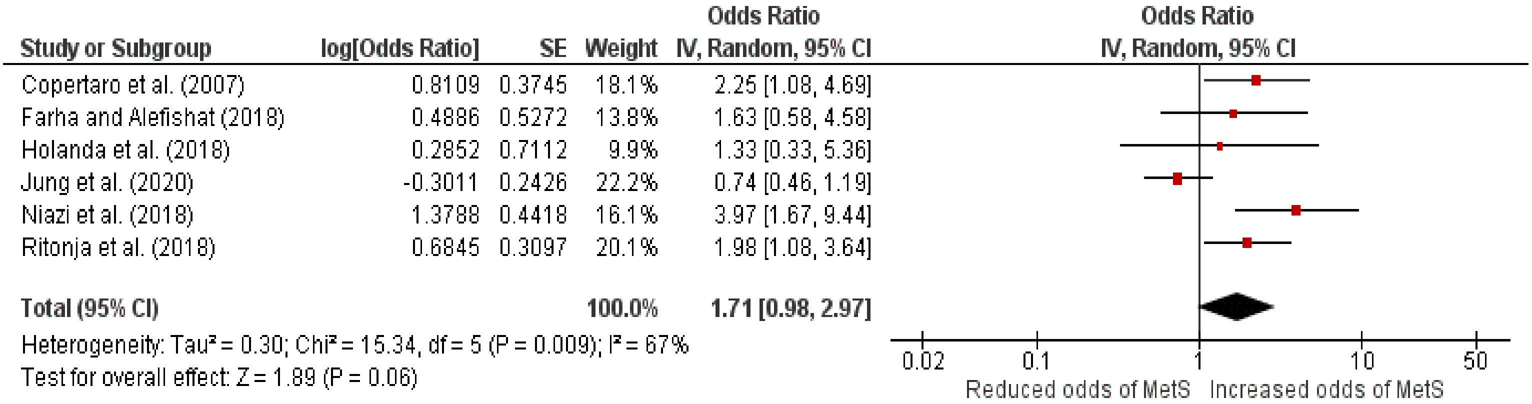


**Figure S2. The pooled estimate of unadjusted ORs**

| Study or Subgroup | log[Odds Ratio] | SE | Weight | Odds Ratio IV, Random, 95% CI |
|---|---|---|---|---|
| Arias et al. (2021) | 3.0969 | 0.4775 | 15.6% | 22.13 [8.68, 56.42] |
| Kirk et al. (2015) | 0.2624 | 0.2606 | 17.9% | 1.30 [0.78, 2.17] |
| Korsiak et al. (2018) | 1.0006 | 0.3462 | 17.1% | 2.72 [1.38, 5.36] |
| Kumar et al. (2021) | -0.5978 | 0.4231 | 16.3% | 0.55 [0.24, 1.26] |
| Lajoie et al. (2015) | 0.8286 | 0.3649 | 16.9% | 2.29 [1.12, 4.68] |
| Pietroiusti et al. (2010) | 1.6292 | 0.4407 | 16.1% | 5.10 [2.15, 12.10] |
| **Total (95% CI)** | | | **100.0%** | **2.74 [1.15, 6.51]** |

Heterogeneity: $Tau^2 = 1.02$; $Chi^2 = 42.19$, df = 5 (P < 0.00001); $I^2 = 88\%$
Test for overall effect: Z = 2.28 (P = 0.02)

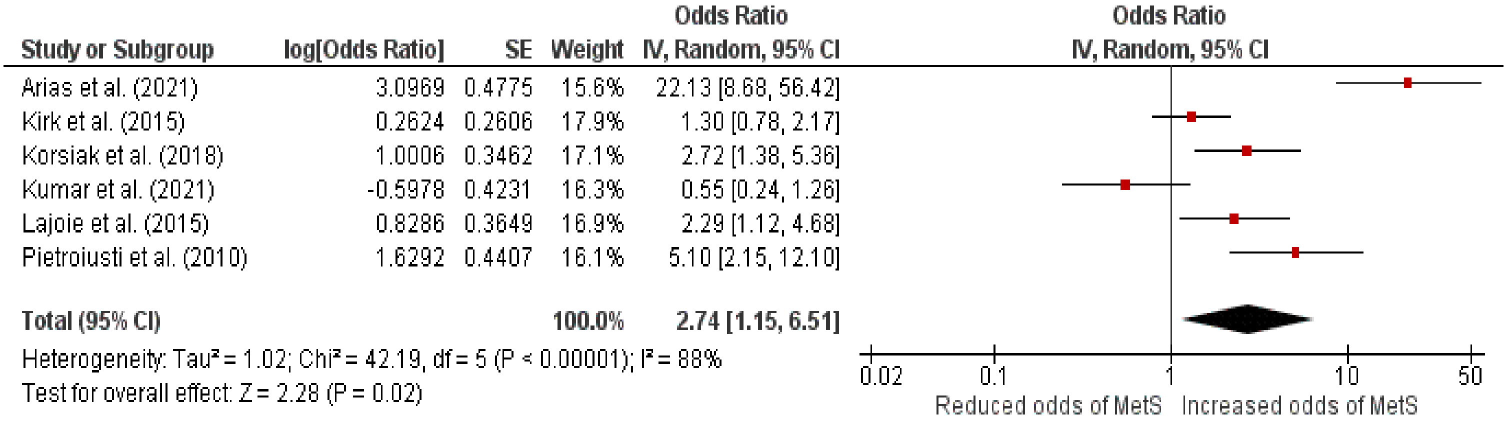


**Figure S3. The pooled estimate of adjusted ORs**

| Study or Subgroup | log[Odds Ratio] | SE | Weight | Odds Ratio IV, Random, 95% CI |
|---|---|---|---|---|
| Copertaro et al. (2007) | 0.8109 | 0.3745 | 17.6% | 2.25 [1.08, 4.69] |
| Holanda et al. (2018) | 0.2852 | 0.7112 | 11.6% | 1.33 [0.33, 5.36] |
| Jung et al. (2020) | -0.3011 | 0.2426 | 20.0% | 0.74 [0.46, 1.19] |
| Korsiak et al. (2018) | 1.0006 | 0.3462 | 18.2% | 2.72 [1.38, 5.36] |
| Niazi et al. (2018) | 1.3788 | 0.4418 | 16.3% | 3.97 [1.67, 9.44] |
| Pietroiusti et al. (2010) | 1.6292 | 0.4407 | 16.4% | 5.10 [2.15, 12.10] |
| **Total (95% CI)** | | | **100.0%** | **2.20 [1.11, 4.38]** |

Heterogeneity: Tau² = 0.56; Chi² = 24.14, df = 5 (P = 0.0002); I² = 79%
Test for overall effect: Z = 2.25 (P = 0.02)

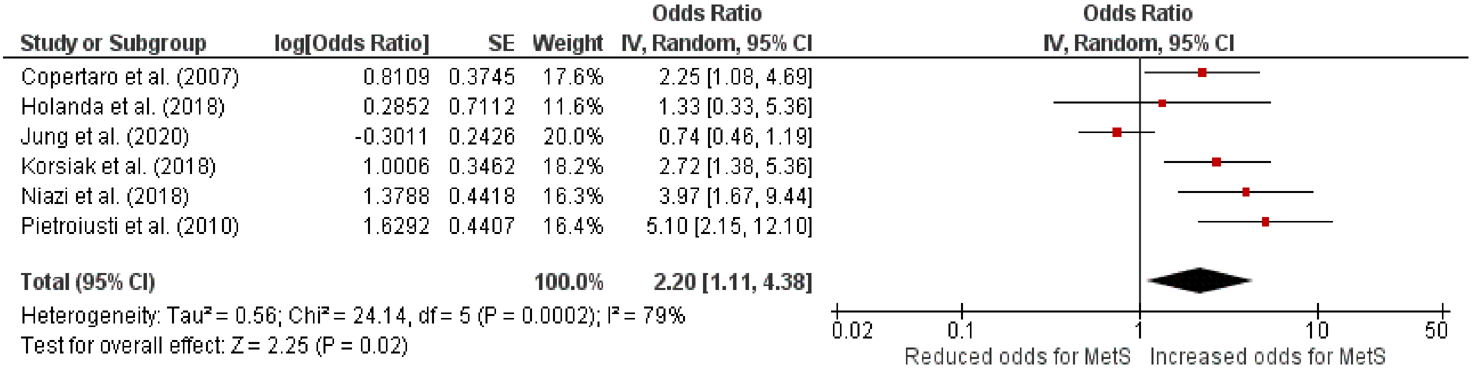


**Figure S4. Pooled risk estimates of night shift work and metabolic syndrome for nurses**

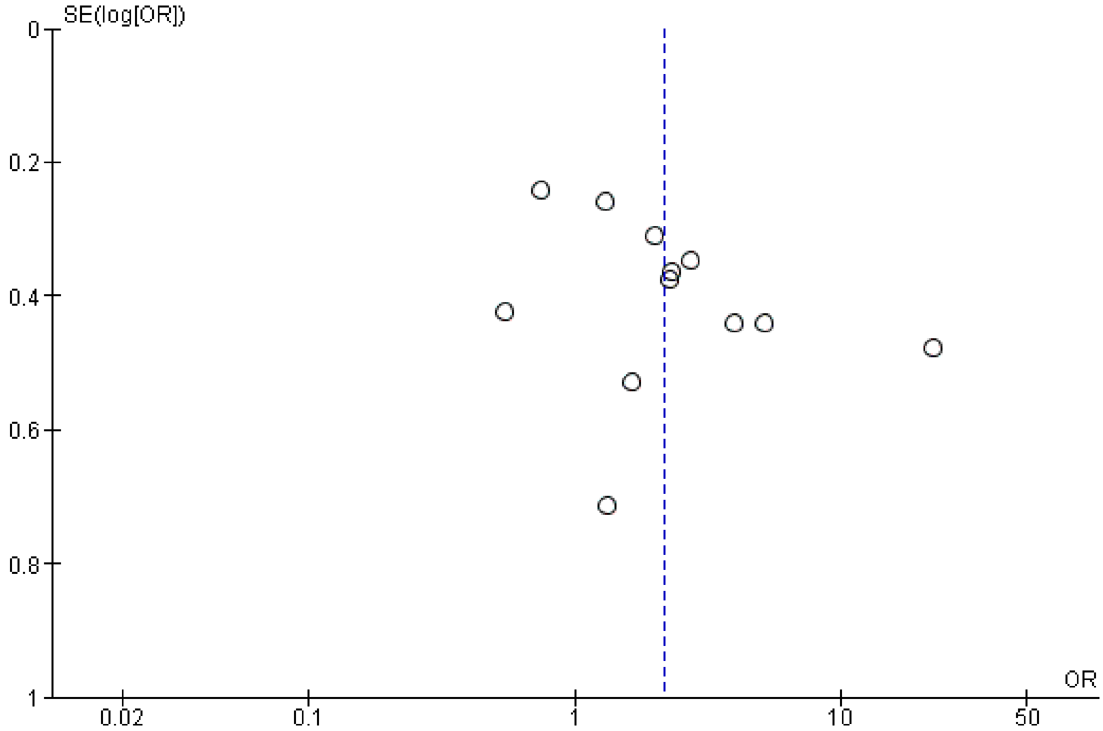


**Figure S5. Funnel plots to assess publication bias**

**Table S1.** Quality analysis

| Study author and year | 1. Was the sample representative of the target population? | 2. Were study participants recruited in an appropriate way? | 3. Was the sample size adequate? | 4. Were the study subjects and setting described in detail? | 5. Was the data analysis conducted with sufficient coverage of the identified sample? | 6. Were valid methods used for the identification of the condition? | 7. Was the condition measured in a standard, reliable way for all participants? | 8. Was there appropriate statistical analysis? | 9. Was the response rate adequate, and if not, was the low response rate managed appropriately? | Total number of "yes" |
|---|---|---|---|---|---|---|---|---|---|---|
| **Arias et al.,2021** | No | Unclear | Yes | Yes | Yes | Yes | Yes | Yes | Yes | **7** |
| **Copertaro et al., 2007** | Yes | Unclear | Unclear | Yes | Yes | Yes | Yes | Yes | Yes | **7** |
| **Farha and Alefishat 2018** | No | Yes | No | Yes | Yes | Yes | Yes | Yes | Yes | **7** |
| **Holanda et al., 2018** | Yes | Yes | No | Yes | Yes | Yes | Yes | Yes | Yes | **8** |
| **Jung et al., 2020** | Yes | Yes | Yes | Yes | Yes | Yes | Yes | Yes | Yes | **9** |
| **Kirk et al., 2015** | No | Yes | Unclear | Yes | Yes | Yes | Yes | Yes | Unclear | **6** |
| **Korsiak et al., 2018** | No | Yes | Unclear | Yes | Yes | Yes | Yes | Yes | Unclear | **6** |
| **Kumar et al., 2021** | Unclear | Yes | Yes | Yes | Yes | Yes | Yes | Yes | Unclear | **7** |
| **Lajoie et al., 2015** | Yes | Yes | Unclear | Yes | Yes | Yes | Yes | Yes | Yes | **8** |
| **Niazi et al., 2018** | Yes | Yes | Unclear | Yes | Yes | Yes | Yes | Yes | Yes | **8** |
| **Pietroiusti et al., 2010** | Yes | Yes | Yes | Yes | Yes | Yes | Yes | Yes | Yes | **9** |
| **Ritonja et al., 2018** | Unclear | Yes | Unclear | Yes | Yes | Yes | Yes | Yes | Yes | **7** |

**Table S2. Recommended policies and interventions to reduce the incidence of MetS among shift workers.**

| **Area** | **Recommended policies and interventions** |
|---|---|
| Work schedules | • Shift schedules should be created using ergonomic criteria that have been proven to reduce stress and limit negative health and well-being impacts by preventing or limiting circadian disturbance and the buildup of sleep deficits and fatigue.<br>• Regulations need to set out limits on maximum shift length, maximum consecutive nights, minimum interval between breaks and weekly and monthly work hours.<br>• Shift schedules should be adjusted to the individual job demands, personal qualities, socioeconomic conditions, and cultural background of the people involved. |
| Healthcare | • Since healthcare employees are subjected to high stress, mental health support programs such as meditation, therapy activities should be organized.<br>• Participating in health screenings covered by benefits, specifically yearly or biannual health screenings for metabolic syndrome and circadian de-synchrony. Before beginning shift work, and at regular intervals later, medical health examinations should be scheduled to assess the compatibility of health issues with shift work |
| Organizational | • Inadequate staffing can result in an excessive individual workload, which may increase the likelihood of work-related stress, error, and health risks for both patients and staff. Therefore, appropriate worker to patient ratios is crucial.<br>• It may be appropriate to nominate one or more employees to assume responsibility for managing the risks connected with shift employment. Broadening their understanding of shift work and familiarizing them with relevant health and safety policy and regulations will aid in the development of a constructive atmosphere for dealing with shift-working arrangements.<br>• Laws and regulations regarding additional rest breaks for meals and naps, supplementary rest-days or holidays to aid recovery, improved canteen facilities and transportation services, health screening, training and rehabilitation courses for shift workers, periodic transfers to day work, and progressive reduction of night work with increasing age could be established. |
| Healthy workplace interventions | • Workplace dietary interventions could be implemented. Shift workers could be given meal replacements with adequate amount of daily nutrition to consume during their busy work hours. |

**Data S1: Search strategy**

Example Database Search Terms: Scopus

( TITLE-ABS-KEY ( "shift work" OR "Shift Work Schedule" OR "Work Schedule Tolerance" OR "Night Shift" OR "night work" OR "irregular working hours" OR "night duty" ) AND TITLE-ABS-KEY ( "Metabolic Syndrome" OR "Dysmetabolic Syndrome" OR "Cardiometabolic Syndrome" OR "Metabolic X Syndrome" OR "Syndrome X" OR "deadly quartet" OR "insulin resistance syndrome" OR "Reaven's Syndrome" ) AND TITLE-ABS-KEY ( "health personnel" OR "health care worker" OR "health worker" OR "care giver" OR "physician" OR "medical staff" OR "nurses" OR "hospital employees" OR "hospital staff" ) AND LANGUAGE ( English ) AND ALL ( "humans" ) AND NOT ALL ( "animals" ) AND NOT ALL ( "animals and humans" ) )

Other databases searched: Pubmed, Web of Science
