# Supplementary material for: Shift work and the risk for metabolic syndrome among healthcare workers: A systematic review and meta‐analysis
Source: Obes Rev. 2022 Jun 22;23(10):e13489. doi: 10.1111/obr.13489 (PMC9539605; doi:10.1111/obr.13489)
Supplement: Supplementary file 3 — Table S1. Quality analysis [file OBR-23-e13489-s002.docx]

| **Study author and year** | **1. Was the sample representative of the target population?** | **2. Were study participants recruited in an appropriate way?** | **3. Was the sample size adequate?** | **4. Were the study subjects and setting described in detail?** | **5.** **Was the data analysis conducted with sufficient coverage of the identified sample?** | **6. Were valid methods used for the identification of the condition?** | **7. Was the condition measured in a standard, reliable way for all participants?** | **8. Was there appropriate statistical analysis?** | **9. Was the response rate adequate, and if not, was the low response rate managed appropriately?** | **Total number of “yes”** |
| --- | --- | --- | --- | --- | --- | --- | --- | --- | --- | --- |
| **Arias et al.,2021** | No | Unclear | Yes | Yes | Yes | Yes | Yes | Yes | Yes | **7** |
| **Copertaro et al., 2007** | Yes | Unclear | Unclear | Yes | Yes | Yes | Yes | Yes | Yes | **7** |
| **Farha and Alefishat 2018** | No | Yes | No | Yes | Yes | Yes | Yes | Yes | Yes | **7** |
| **Holanda et al., 2018** | Yes | Yes | No | Yes | Yes | Yes | Yes | Yes | Yes | **8** |
| **Jung et al., 2020** | Yes | Yes | Yes | Yes | Yes | Yes | Yes | Yes | Yes | **9** |
| **Kirk et al., 2015** | No | Yes | Unclear | Yes | Yes | Yes | Yes | Yes | Unclear | **6** |
| **Korsiak et al., 2018** | No | Yes | Unclear | Yes | Yes | Yes | Yes | Yes | Unclear | **6** |
| **Kumar et al., 2021** | Unclear | Yes | Yes | Yes | Yes | Yes | Yes | Yes | Unclear | **7** |
| **Lajoie et al., 2015** | Yes | Yes | Unclear | Yes | Yes | Yes | Yes | Yes | Yes | **8** |
| **Niazi et al., 2018** | Yes | Yes | Unclear | Yes | Yes | Yes | Yes | Yes | Yes | **8** |
| **Pietroiusti et al., 2010** | Yes | Yes | Yes | Yes | Yes | Yes | Yes | Yes | Yes | **9** |
| **Ritonja et al., 2018** | Unclear | Yes | Unclear | Yes | Yes | Yes | Yes | Yes | Yes | **7** |
