# Supplementary material for: Shift work and the risk for metabolic syndrome among healthcare workers: A systematic review and meta‐analysis
Source: Obes Rev. 2022 Jun 22;23(10):e13489. doi: 10.1111/obr.13489 (PMC9539605; doi:10.1111/obr.13489)
Supplement: Supplementary file 4 — Table S2. Recommended policies and interventions to reduce the incidence of MetS among shift workers. [file OBR-23-e13489-s004.docx]

**Table S2.** Recommended policies and interventions to reduce the incidence of MetS among shift workers.

| **Area** | **Recommended policies and interventions** |
| --- | --- |
| Work schedules | - Shift schedules should be created using ergonomic criteria that have been proven to reduce stress and limit negative health and well-being impacts by preventing or limiting circadian disturbance and the buildup of sleep deficits and fatigue. - Regulations need to set out limits on maximum shift length, maximum consecutive nights, minimum interval between breaks and weekly and monthly work hours. - Shift schedules should be adjusted to the individual job demands, personal qualities, socioeconomic conditions, and cultural background of the people involved. |
| Healthcare | - Since healthcare employees are subjected to high stress, mental health support programs such as meditation, therapy activities should be organized. - Participating in health screenings covered by benefits, specifically yearly or biannual health screenings for metabolic syndrome and circadian de-synchrony. Before beginning shift work, and at regular intervals later, medical health examinations should be scheduled to assess the compatibility of health issues with shift work |
| Organizational | - Inadequate staffing can result in an excessive individual workload, which may increase the likelihood of work-related stress, error, and health risks for both patients and staff. Therefore, appropriate worker to patient ratios is crucial. - It may be appropriate to nominate one or more employees to assume responsibility for managing the risks connected with shift employment. Broadening their understanding of shift work and familiarizing them with relevant health and safety policy and regulations will aid in the development of a constructive atmosphere for dealing with shift-working arrangements. - Laws and regulations regarding additional rest breaks for meals and naps, supplementary rest-days or holidays to aid recovery, improved canteen facilities and transportation services, health screening, training and rehabilitation courses for shift workers, periodic transfers to day work, and progressive reduction of night work with increasing age could be established. |
| Healthy workplace interventions | - Workplace dietary interventions could be implemented. Shift workers could be given meal replacements with adequate amount of daily nutrition to consume during their busy work hours. |
